# Adverse events associated with colonoscopy; an examination of online concerns

**DOI:** 10.1186/s12876-019-1127-5

**Published:** 2019-12-03

**Authors:** Elad Yom-Tov, Benjamin Lebwohl

**Affiliations:** 1 Microsoft Research, Herzeliya, Israel; 2 Faculty of Industrial Engineering and Management, Technion – Israel Institute of Technology, Haifa, Israel; 3 Celiac Disease Center at Columbia University, New York, USA

**Keywords:** Colonoscopy, Colorectal Cancer, Complications

## Abstract

**Background:**

Colonoscopy as a screening and diagnostic tool is generally safe and well-tolerated, and significant complications are rare. The rate of more mild adverse effects is difficult to estimate, particularly when such effects do not result in hospital admission. We aimed to identify the rate and timing of adverse effects as reported by users querying symptoms on an internet search engine.

**Methods:**

We identified queries made to Bing originating from users in the United States containing the word “colonoscopy” during a 12-month period and identified those queries in which the timing of colonoscopy could be estimated. We then identified queries from those same users for medical symptoms during the time span from 5 days before through 30 days after the colonoscopy date.

**Results:**

Of 641,223 users mentioning colonoscopy, 7013 (1.1%) had a query that enabled identification of their colonoscopy date. The majority of queries about colonoscopy preceded the procedure, and concerned diet. 28% of colonoscopy-related queries were made afterwards, and included queries about diarrhea and cramps, with 2.6% of users querying respiratory symptoms after the procedure, including cough (1.2%) and pneumonia (0.6%). Respiratory symptoms rose significantly at days 7–10 after the colonoscopy.

**Conclusions:**

Internet search queries for respiratory symptoms rose approximately one week after queries relating to colonoscopy, raising the possibility that such symptoms are an under-reported late adverse effect of the procedure. Given the widespread use of colonoscopy as a screening modality and the rise of anesthesia-assisted colonoscopy in the United States in recent years, this signal is of potential public health concern.

## Background

Colonoscopy is the most commonly used method for colorectal cancer screening in the United States, and nearly 60% of individuals eligible for colorectal cancer screening have undergone this procedure [1]. Among individuals undergoing alternative modes of screening, such as fecal immunochemical testing, colonoscopy is mandated for those who have a positive screening test, and colonoscopy is the effector arm of all colorectal cancer screening tests [2].

Given its widespread use in the general population, the effectiveness of colonoscopy is dependent on its safety profile, particularly when it is employed as a primary screening modality in asymptomatic individuals. Numerous studies have confirmed that the rate of serious complications after colonoscopy is low, and that the most severe complications of the procedure (perforation, bleeding, and mortality) have declined in recent years [3]. However, respiratory complications, such as aspiration pneumonia, may be an underappreciated sequela of colonoscopy, particularly when deep sedation with anesthesia assistance is provided [4, 5, 6]. As rates of anesthesia assistance during colonoscopy have increased markedly in recent years [7, 8], there is concern that respiratory complications may be an increasingly common event.

Prior studies of colonoscopy complications focused on the severe end of the spectrum, and mostly relied on post-colonoscopy emergency department visits or hospitalizations to identify complications [3, 4, 9–14]. Such methods likely underestimate more mild, but still clinically significant events that do not meet the threshold for acute care, such as a febrile respiratory illness with cough. These events are difficult to measure using traditional measures, but may be estimated using search engine queries [15]. Such a strategy has been used to estimate the prevalence of symptoms that did rise to the threshold of seeking care, but that preceded a diagnosis of adenocarcinoma of the pancreas [16]. 

In this study we aimed to determine the prevalence and timing of post-colonoscopy complications using search engine queries.

## Methods

### Data

We extracted all queries made to Bing from people in the USA between October 1st, 2017 and September 30th, 2018 (one year). Each query comprised of an anonymized user identifier, the time and date of the query, and its text. The queries were filtered to include those queries which contained the word “colonoscopy” and any query made by people who searched for this word. The age and gender of users, as provided by users when they registered with Bing, was available for a subset (23%) of the users.

### Timing of colonoscopy

Queries that mentioned colonoscopy were filtered to include those that mentioned a reference date. These queries included text in the form of “colonoscopy in X days,” “colonoscopy X days ago,” “colonoscopy tomorrow,” or “colonoscopy yesterday.” These queries allowed us to pinpoint the date on which colonoscopies occurred for each user. The time of each query was normalized to the calculated date of colonoscopy.

### Adverse events

Other queries made by people for whom colonoscopies could be timed were filtered to include those queries which mentioned one or more of 195 medical symptoms and their synonyms [17]. This list was augmented with terms related to fainting, pneumonia, and bronchitis, as well as with a list of common antibiotics.

We calculated the number of people who queried for each of the symptoms as a function of time, relative to the colonoscopy date, between 5 days before the date of colonoscopy and until 30 days after it. This number was first normalized to the probability of querying for the symptom over the entire time range. In line with previous work [18], we then normalized this probability by the probability of a user who underwent colonoscopy to ask about any symptom on each day. Finally, we filtered the time series using a 3-day moving average. We refer to this time series as the symptom ratio time series (SRTS). SRTS were retained for those symptoms which were queried for by 75 people or more. To calculate significant SRTS values we found the 5% highest values of SRTS among all SRTSs.

## Results

**Fig. 1:**
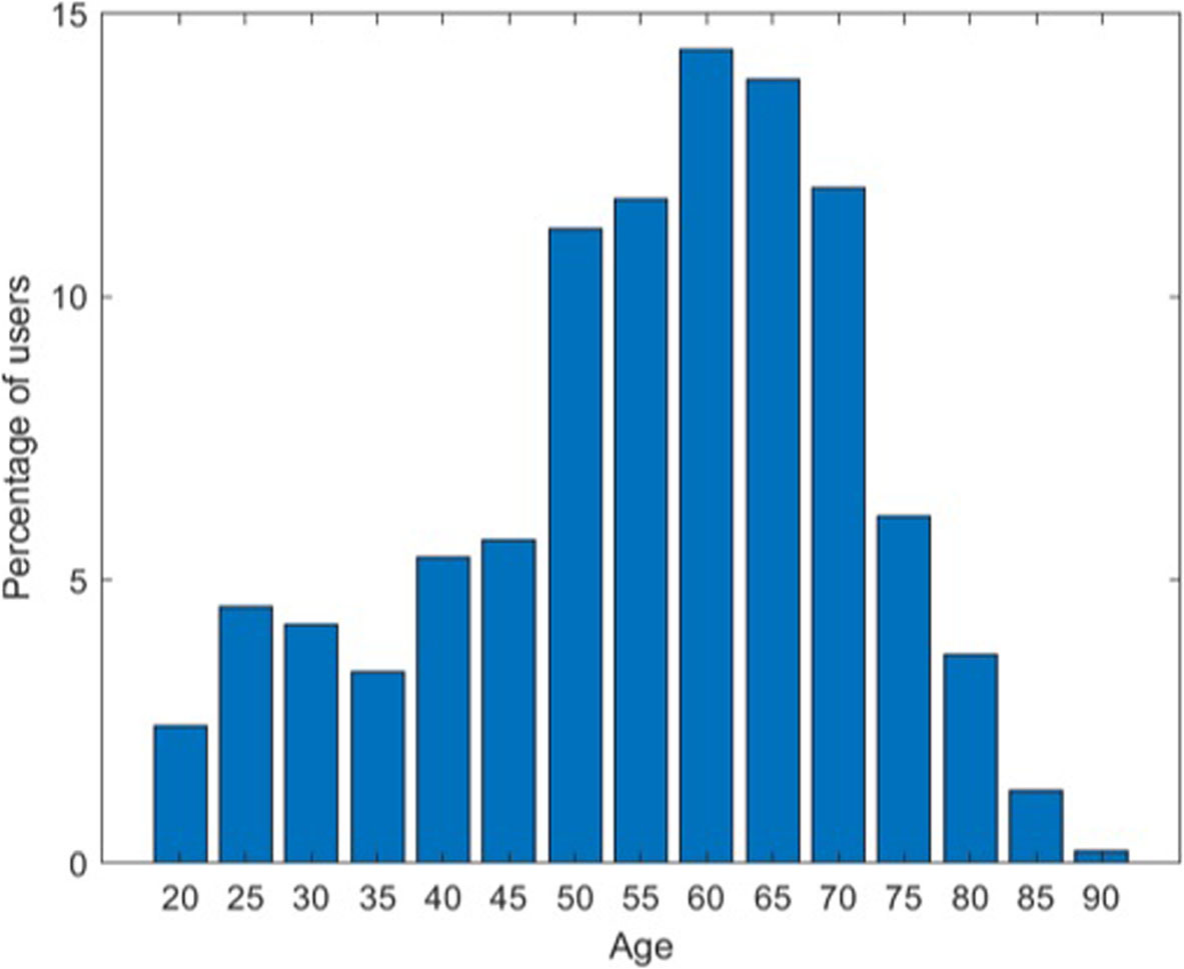
Age distribution of users undergoing colonoscopy

During the 12-month period, 641,223 people mentioned colonoscopy in their queries. Among them, a total of 7013 users made queries that enabled identification of their colonoscopy date. Females accounted for 60.0% of users in our data. Figure 1 shows the age distribution of the 947 people who underwent colonoscopy for whom age data were available. The median age was 58 years, and the most common age group was 60–64 years.

**Fig. 2:**
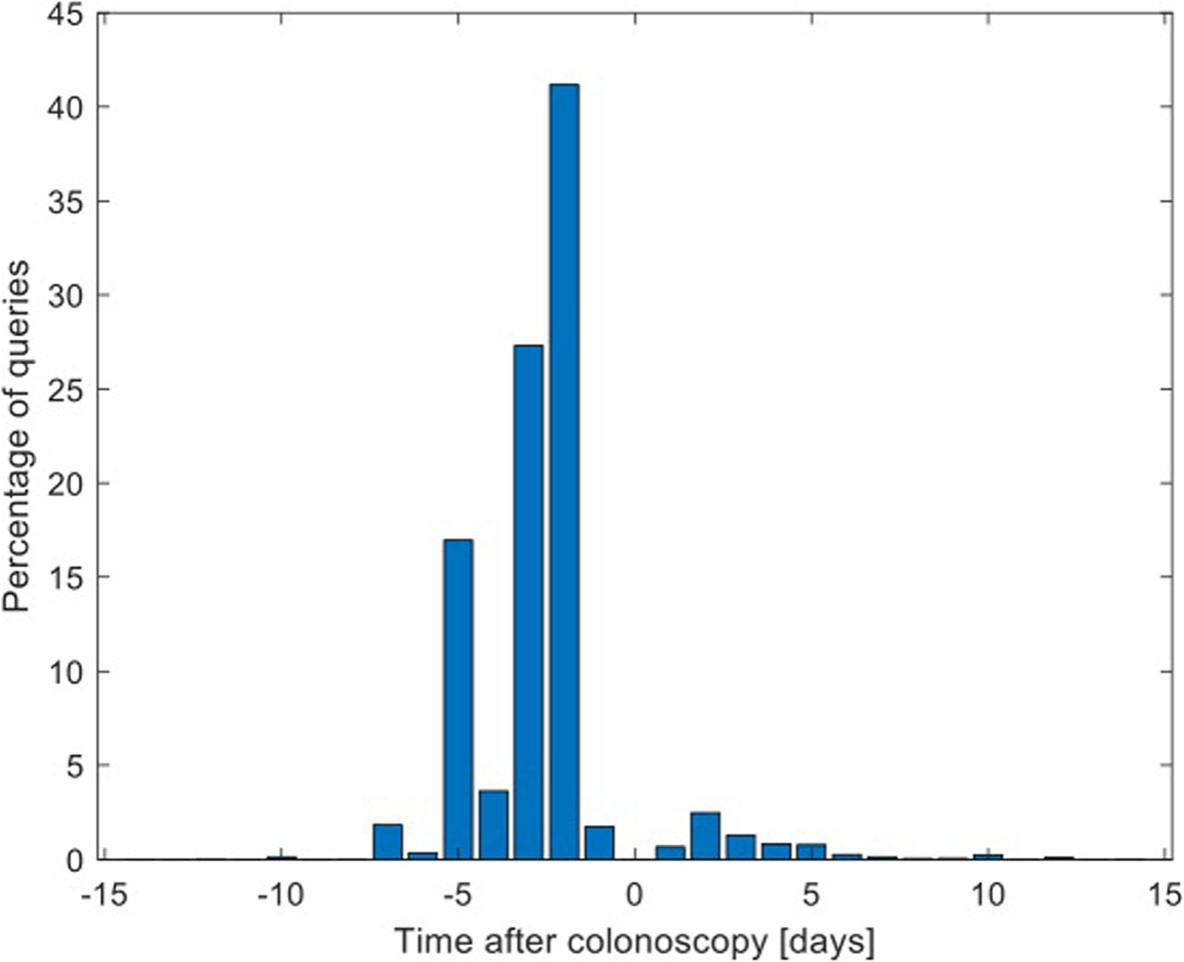
Distribution of queries which mentioned the time relative to colonoscopy. Positive times indicate that the query was made after the colonoscopy

Figure 2 shows the distribution of queries by 7013 who mentioned time relative to the colonoscopy date, where positive numbers indicated that the query was made after the colonoscopy (e.g., “I had colonoscopy yesterday”). The figure shows that many more people ask about issues related to colonoscopy before the procedure than after it.

Table 1 shows the most common words and word pairs before and after the procedure, excluding stopwords [19], words about the number of days relative to colonoscopy, or the word “colonoscopy.” The table also shows the most common queries before and after the procedure. As the table demonstrates, concerns prior to colonoscopy include mostly the diet required before it, while queries after the procedure refer mostly to adverse events associated with colonoscopy.

**Table 1:** Most common words, word pairs, and queries before and after colonoscopies. Day numbers were replaced by “X”

Before the procedure	After the procedure
Words and word pairs	
Eat	Pain
Diet	Normal
To eat	Still
Foods	Blood
Prior	Stool
Queries	
diet X days before colonoscopy	diarrhea X days after colonoscopy
what to eat X days before colonoscopy	no bowel movement X days after colonoscopy
colonoscopy diet X days before	stomach cramps X days after colonoscopy
X days before colonoscopy diet	bloody stool X days after colonoscopy
X days before colonoscopy	cramps X days after colonoscopy

### Adverse reactions

**Fig. 3:**
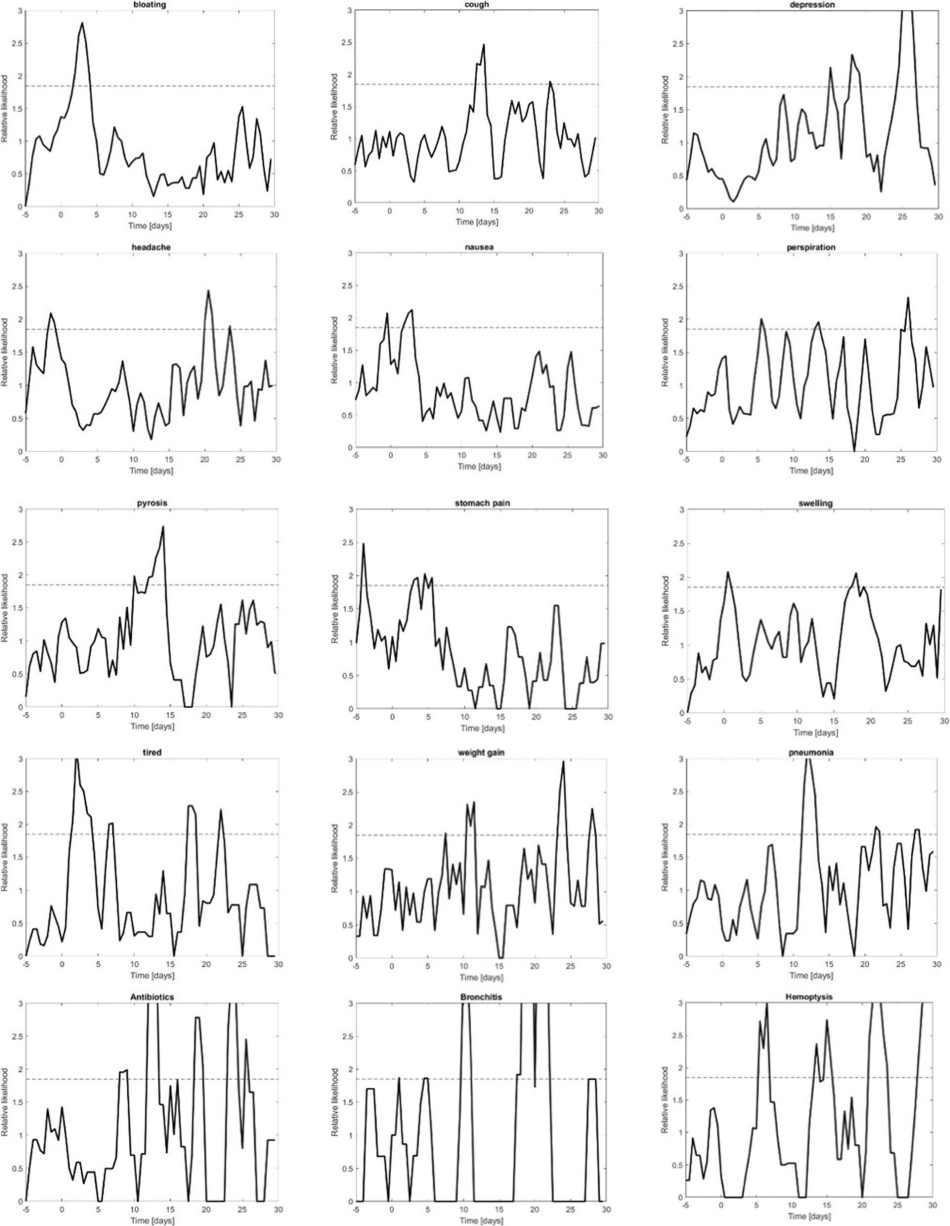
Symptom ratio time series (SRTS) with statistically significant values over more than three days. The dotted line shows the critical value for statistical significance

Of all symptom queries made in the peri-procedure period, 28% were made after the procedure. Figure 3 shows the 15 symptoms, conditions and drugs that had more than three days with statistically significant SRTS values. Table 2 shows the percentage of users (*n* = 7013) who asked about each of the terms in Fig. 3 after the colonoscopy. As the figure shows, for some symptoms tend to cluster during the first few days after colonoscopy (bloating, nausea, stomach pain and tiredness). However, other symptoms exhibit statistically significant high values of SRTS in later days. These include respiratory symptoms such as cough and queries for pneumonia, as well as fever, headaches, and weight gain.

**Table 2:** Percentage of users (and 95% confidence intervals) who queried for symptoms, drugs, and conditions after colonoscopy (n = 7013). Also shown are the average age of users and the percentage of females

Term	Percentage	95% CI	Average age (s.e.)	Percent female
Bloating	1.28	[1.03–1.58]	52.8 (10.6)	72.7
Depression	1.08	[0.86–1.35]	50.5 (17.5)	47.8
Headache	1.04	[0.81–1.31]	58.7 (12.4)	57.1
Nausea	0.88	[0.68–1.13]	47.7 (14.2)	75.0
Perspiration	1.25	[1.01–1.54]	55.9 (16.9)	47.4
Fever	0.66	[0.74–1.21]	53.6 (15.5)	43.5
Stomach pain	0.71	[0.41–0.80]	56.9 (19.1)	50.0
Swelling	1.13	[0.90–1.40]	50.9 (15.4)	50.0
Tired	0.88	[0.68–1.13]	51.2 (18.0)	43.8
Weight gain	0.70	[0.51–0.93]	51.2 (19.9)	57.9
Any respiratory symptom	2.57	[2.21–2.97]	50.5 (18.7)	64.5
Cough	1.21	[1.03–1.58]	49.5 (20.3)	80.0
Pneumonia	0.58	[0.41–0.80]	44.3 (16.0)	75.0
Antibiotics	0.33	[0.23–0.50]	43.6 (22.5)	80.0
Bronchitis	0.19	[0.10–0.31]	62.0 (5.0)	100.0
Hemoptysis	0.44	[0.30–0.63]	55.7 (17.4)	14.3

Table 2 also shows the average age and percent of females who asked about each symptom. Interestingly, females ask more about respiratory symptoms (with the exception of hemoptysis), while the general symptoms are slightly more common among males, with the exception of bloating and nausea.

## Discussion

In this analysis of colonoscopy-related internet search queries, we found that queries were far more common before the procedure than after the procedure, and that pre-procedure diet was the most commonly queried subject. But we also found that a substantial minority of patients (28%) query symptoms in the days following colonoscopy, and that there was a measurable increase in queries related to respiratory symptoms, suggesting that aspiration during the procedure may be an under-reported adverse effect of colonoscopy.

Colonoscopy has been associated with a reduced mortality from colorectal cancer in case-control and cohort studies [20–22], and the risk of post-colonoscopy colorectal cancer has been linked to a measure of colonoscopy quality, the adenoma detection rate [23]. The decrease in colorectal cancer incidence in the United States in recent decades can be attributed in part to the uptake in screening for colorectal cancer, in which precancerous adenomas are identified and removed during colonoscopy [24, 25]. At the same time, there is evidence that colonoscopy may be overused in some contexts, in which patients undergo the procedure more frequently than recommended by guidelines [26, 27], undermining cost-effectiveness and exposing patients to risks without a clear incremental benefit.

As colonoscopy is a frequently-performed procedure on a large proportion of the population, it is particularly relevant to identify relatively mild symptoms that may not reach the threshold of detection using claims-based analyses that rely on health care encounters. Such studies have found that respiratory complications are rare. For instance, a population-based cohort study of the 5% sample of cancer-free Medicare beneficiaries in SEER-Medicare regions found that hospitalization for aspiration was the most frequent complication of colonoscopy, but occurred in less than 0.2% cases [6]. Similarly, an analysis of Health Care Cost and Utilization Project data from California on screening and surveillance colonoscopy performed between 2005 and 2011 (*n* = 1.58 million) found that the rate of emergency department visits or hospital admissions for pulmonary complications within 30 days following the procedure was 31 per 10,000 (0.3%) [10]. Our study, that found a pulmonary symptom rate of 2.6%, suggests that the previously documented rare risk of aspiration represents the most severe end of the spectrum of such symptoms, and that more mild symptoms are under-reported yet present.

The finding of a respiratory signal peaking one week after colonoscopy is of particular concern given the secular trend of increasing use of anesthesia assistance during this procedure [7, 8, 28]. Although the use of anesthesia assistance is associated with increased patient satisfaction and improved throughput, two large studies in the United States found a small but statistically significant increase in the risk of aspiration pneumonia following colonoscopy with anesthesia assistance as compared to those utilizing conscious sedation [4, 5].

To our knowledge, this is the first study to identify self-reported symptoms after colonoscopy using internet queries. Such a strategy has been used to identify symptoms compatible with respiratory syncytial virus, and has tracked with CDC-monitored rates [29]. Though the methodology and findings are novel, we acknowledge that this study has a number of limitations. We were unable to confirm the veracity of these estimates for colonoscopy dates via medical record review; a user querying diet before colonoscopy symptoms after a colonoscopy may be querying on behalf of a friend or relative, or may be asking such questions hypothetically. Selection bias is also a concern. Only a small proportion (1.1%) of users who queried colonoscopy had an estimatable date of the procedure based on the query, and of such users, only 28% queried any symptoms afterward. Users who queried any symptoms may be different in important ways from users who did not query symptoms after colonoscopy.

## Conclusion

We found that internet search queries for respiratory symptoms rose approximately one week after queries relating to colonoscopy, raising the possibility that such symptoms are an under-reported late adverse effect of the procedure. Given the widespread use of colonoscopy as a screening modality and the rise of anesthesia-assisted colonoscopy in the United States in recent years, this signal is of potential public health concern. Future studies should focus on measuring the incidence of and risk factors for cough and other respiratory symptoms following colonoscopy, recording the type of sedation used, employing a prospectively administered questionnaire, and including a non-colonoscopy control group. Such as study, designed to identify symptoms that do not necessarily reach the threshold for hospitalization, would clarify the potential safety signal identified in this analysis.

## Data Availability

All data relevant to the study are included in the article. The data that support the findings of this study are available from Microsoft, but restrictions apply to the availability of the data. Individual-level search data are available from the authors on reasonable request and with permission of Microsoft.

## Abbreviations

CDC: Centers for Disease Control
SEER: Surveillance Epidemiology and End Results program
STRS: Symptom ratio time series
